# Selection and Application of Tissue microRNAs for Nonendoscopic Diagnosis of Barrett’s Esophagus

**DOI:** 10.1053/j.gastro.2018.05.050

**Published:** 2018-09

**Authors:** Xiaodun Li, Sam Kleeman, Sally B. Coburn, Carlo Fumagalli, Juliane Perner, Sriganesh Jammula, Ruth M. Pfeiffer, Linda Orzolek, Haiping Hao, Philip R. Taylor, Ahmad Miremadi, Núria Galeano-Dalmau, Pierre Lao-Sirieix, Maria Tennyson, Shona MacRae, Michael B. Cook, Rebecca C. Fitzgerald

**Affiliations:** 1 MRC Cancer Unit, Hutchison-MRC Research Centre, University of Cambridge, Cambridge, UK; 2 Division of Cancer Epidemiology and Genetics, National Cancer Institute, National Institutes of Health, Department of Health and Human Services, Rockville, Maryland; 3 Cancer Research UK Cambridge Institute, University of Cambridge, Cambridge, UK; 4 Johns Hopkins Medical Institutions Deep Sequencing and Microarray Core, Baltimore, Maryland; 5 Cambridge University Hospitals NHS Trust, Cambridge UK

**Keywords:** Gene Regulation, Biomarker, Esophageal Adenocarcinoma, Diagnosis, AUC, area under the curve, BE, Barrett’s esophagus, BNE, squamous epithelium from above the Barrett’s segment, CI, confidence interval, FFPE, formalin-fixed, paraffin-embedded, mRNA, messenger RNA, miRNA, microRNA, NE, normal esophagus, NES, normal esophageal squamous, TFF3, trefoil factor 3, UTR, untranslated region

## Abstract

**Background & Aims:**

MicroRNA (miRNA) is highly stable in biospecimens and provides tissue-specific profiles, making it a useful biomarker of carcinogenesis. We aimed to discover a set of miRNAs that could accurately discriminate Barrett’s esophagus (BE) from normal esophageal tissue and to test its diagnostic accuracy when applied to samples collected by a noninvasive esophageal cell sampling device.

**Methods:**

We analyzed miRNA expression profiles of 2 independent sets of esophageal biopsy tissues collected during endoscopy from 38 patients with BE and 26 patients with normal esophagus (controls) using Agilent microarray and Nanostring nCounter assays. Consistently up-regulated miRNAs were quantified by real-time polymerase chain reaction in esophageal tissues collected by Cytosponge from patients with BE vs without BE. miRNAs were expressed from plasmids and antisense oligonucleotides were expressed in normal esophageal squamous cells; effects on proliferation and gene expression patterns were analyzed.

**Results:**

We identified 15 miRNAs that were significantly up-regulated in BE vs control tissues. Of these, 11 (MIR215, MIR194, MIR 192, MIR196a, MIR199b, MIR10a, MIR145, MIR181a, MIR30a, MIR7, and MIR199a) were validated in Cytosponge samples. The miRNAs with the greatest increases in BE tissues (7.9-fold increase in expression or more, *P* < .0001: MIR196a, MIR192, MIR194, and MIR215) each identified BE vs control tissues with area under the curve (AUC) values of 0.82 or more. We developed an optimized multivariable logistic regression model, based on expression levels of 6 miRNAs (MIR7, MIR30a, MIR181a, MIR192, MIR196a, and MIR199a), that identified patients with BE with an AUC value of 0.89, 86.2% sensitivity, and 91.6% specificity. Expression level of MIR192, MIR196a, MIR199a, combined that of trefoil factor 3, identified patients with BE with an AUC of 0.93, 93.1% sensitivity, and 93.7% specificity. Hypomethylation was observed in the promoter region of the highly up-regulated cluster MIR192–MIR194. Overexpression of these miRNAs in normal esophageal squamous cells increased their proliferation, via GRHL3 and PTEN signaling.

**Conclusions:**

In analyses of miRNA expression patterns of BE vs non-BE tissues, we identified a profile that can identify Cytosponge samples from patients with BE with an AUC of 0.93. Expression of MIR194 is increased in BE samples via epigenetic mechanisms that might be involved in BE pathogenesis.

## Graphical abstract

**Figure:**
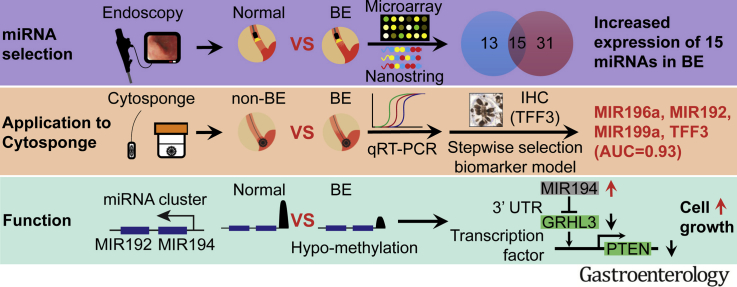

See Covering the Cover synopsis on page 585.

**What You Need to Know**

**Background and Context**

microRNAs are a biomarker for cancer and some data supports differential expression in Barrett's esophagus and esophageal adenocarcinoma.

**New Findings**

By applying dual discovery platforms this study identified a panel of upregulated microRNAs that could discriminate BE from normal esophagus in endoscopic biopsies as well as in samples collected by a non-endoscopic BE diagnosis device (Cytosponge^®^). Laboratory experiments suggest that increased expression of the upregulated MIR 192-194 cluster causes increased proliferation via altered signaling pathways.

**Limitations**

The miRNA panel requires further validation on an independent set of Cytosponge^®^ samples.

**Impact**

A panel of differentially expressed microRNAs could be used to diagnose BE patients using a minimally invasive cell collection device, thus reducing the burden on endoscopy.

Esophageal adenocarcinoma is a highly lethal malignancy with a 5-year survival of less than 20%.^1^ Although the precursor metaplasia, Barrett’s esophagus (BE), provides an opportunity for surveillance and early detection, 95% of patients with esophageal adenocarcinoma are diagnosed in individuals without a prior diagnosis of BE.^2^, ^3^ This conundrum necessitates the development of new strategies and tools to identify a larger proportion of individuals who have BE.

Any potentially useful screening tool for BE needs to be highly sensitive (to avoid harm caused by false-negatives), highly specific (to avoid financial costs of conducting unnecessary secondary investigations), and logistically feasible and affordable to be suitable on a large scale. A minimally invasive, pan-esophageal cell sampling device, the Cytosponge, coupled with immunohistochemical staining for Trefoil Factor 3 (TFF3), has been shown to have cost-effective utility in diagnosing BE^4^ with applicability to the primary care setting.^5^ In the BEST2 case-control study, there was encouraging sensitivity and specificity (sensitivity 79.9% for all segment lengths, using an “intention-to-treat” analysis whereby samples lacking columnar cells, indicating that the Cytosponge did not reach the stomach, are included; specificity 92.4%).^6^ As most patients with BE will not progress to cancer, we have investigated additional nucleic acid biomarkers for the Cytosponge, including methylation and p53 mutations to stratify patients according to their risk of progression to cancer.^7^, ^8^ Ideally, a single automated platform using nucleic acids (DNA and RNA) extracted from the Cytosponge could be used to diagnose and risk-stratify patients in parallel rather than relying on a 2-step process involving an immunohistochemical biomarker.

MicroRNAs (miRNAs) are a type of small (18–22 nucleotides) non–protein-coding RNA that bind to messenger RNAs (mRNAs) via their seed sequence to repress gene expression post-transcriptionally.^9^, ^10^ miRNAs are found across the genome, sometimes within introns of genes, and clusters of miRNA loci are commonly observed. Regulatory elements that control transcription are usually shared within an miRNA cluster or with neighbouring genes, although the latter is under a more complex modulation.^11^ miRNAs have central roles in endogenous processes, including metabolism, inflammation, and carcinogenesis, and each miRNA has the potential to regulate a diverse array of gene transcripts.^12^ miRNA profiles have been shown to be tissue and disease specific^13^ and are minimally affected by processes used to generate formalin-fixed, paraffin-embedded (FFPE) samples.^14^, ^15^ These features of miRNAs make them appealing biomarkers. Previous studies have identified several candidate miRNA biomarkers specific for BE; however, these were limited by relatively small sample sizes, lack of any functional validation, and reliance on a single profiling platform that constrained the diversity of miRNAs that are quantified.^16^, ^17^, ^18^, ^19^, ^20^, ^21^ Furthermore, we were particularly interested to test the application of an accurate miRNA classifier to Cytosponge samples for the purposes of diagnosing BE.

The aims of the study were (1) to discover a miRNA signature that could distinguish BE from normal esophagus (NE) across 2 distinct profiling platforms; (2) to validate this miRNA signature using a Cytosponge case-control sample set; and (3) to examine the functional consequences of the most up-regulated miRNAs in vitro.

## Methods

### Sample Selection

**Figure 1:**
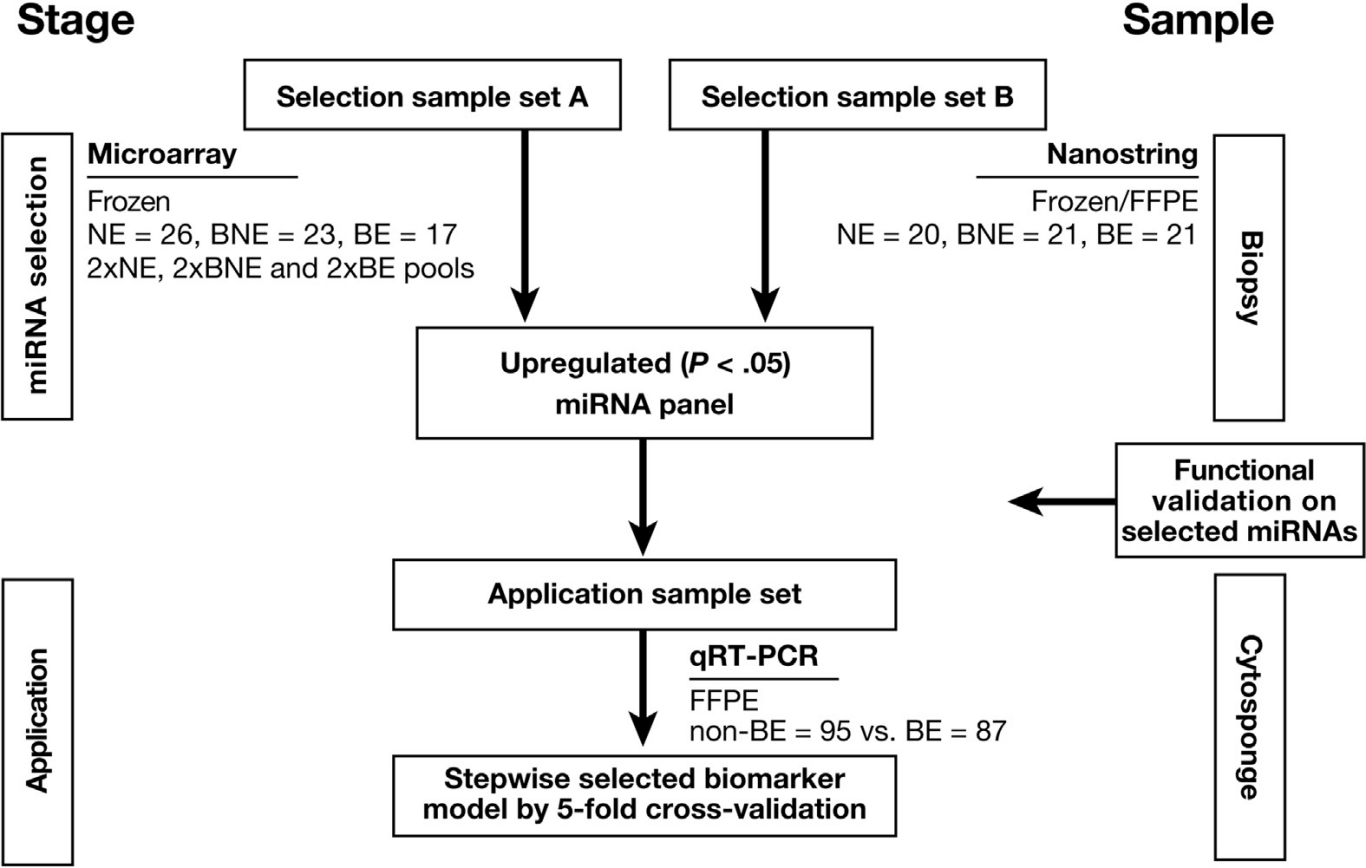
Schematic illustration of the study design and strategy. Summary of methods divided into 2 stages of Selection (A and B) and application using both biopsy-derived and Cytosponge-derived samples.

The samples for the different parts of the study are summarized in Figure 1. Patients in sample set A were from an ongoing prospective Barrett’s biobank (Ethics No. LREC 01/149) and sample set B from endoscopic samples collected as part of BEST1 (Ethics No. 06/Q0108/272) and BEST2 (Ethics No. 10/H0308/71). There was no overlap between the 2 sample sets. All samples were obtained following ethical approval and individual informed consent.

Sample sets A and B comprised cases and controls and for sample set A pools were created. Cases comprised patients with a known diagnosis of BE attending for surveillance and controls were individuals referred to endoscopy because of dyspepsia and/or reflux symptoms. All biopsy samples were subject to an expert histopathological review before inclusion. NE biopsy samples contained stratified squamous epithelium and an absence of columnar cells. BE biopsy samples contained intestinal metaplasia without dysplasia or neoplasia.

The up-regulated miRNAs were then tested in Cytosponge samples. These samples were randomly selected from the BEST2 comprising cases (BE) and controls (individuals referred for endoscopy because of dyspepsia or reflux symptoms with BE). The Cytosponge sample had to have sufficient material remaining for miRNA analysis to be included.

### miRNA Extraction

RNA from frozen regular forceps biopsy samples was extracted using the miRNeasy Mini Kit (Qiagen, Valencia, CA) according to the manufacturer’s protocol. For FFPE blocks, 2 to 4 scrolls of 10 mm were cut and extracted using the miRNeasy FFPE Kit (Qiagen) according to the manufacturer’s protocol. Total RNA concentrations were measured by ND-1000 spectrophotometer (NanoDrop Technologies, Wilmington, DE) and 2100 Bioanalyzer (Agilent Technologies, Santa, Clara, CA).

### Microarray Expression Analysis

miRNA microarray was performed with the Human miRNA Microarray kit v1.0 (8 × 15 K, consists of 534 probes of 470 human and 64 virus mature miRNAs) (Agilent Technologies) with 100 ng total RNA per sample per the manufacturer’s protocol. The hybridized chip was scanned using the G2565BA Microarray Scanner (Agilent Technologies) and analyzed using GenePix Pro software v4.1 (Molecular Devices Corporation, San Jose, CA). Platform annotations were re-annotated to miRBase 21.0 using miRiadne.^22^ Raw intensities were then log_2_-transformed and normalized by quantile with differentially expressed miRNAs identified using linear models implemented in the limma package (version 3.32.2) for R (version 3.4.0).^23^ We filtered any miRNAs with fewer than 2/6 pools expressing at above 6.5 (corresponding to intensity above 90) and if multiple probes mapped to the same miRNA, the probe with the highest average expression was selected.^24^

### Nanostring nCounter Analysis

Samples were sent to Johns Hopkins Medical Institute Deep Sequencing and Core Facility for NanoString nCounter analysis. RNA samples were processed according to the manufacturer’s protocol for the nCounter Human miRNA Expression Assay v2 kit, which profiled 800 human miRNAs (NanoString, Seattle, WA). We used 100 ng of each total RNA sample as input into the nCounter Human miRNA sample preparation. Hybridization with the capture probe set was incubated for 16 hours. Counts were collated for each sample by the nCounter Digital Analyzer and raw counts were imported into nSolver version 3.0.

Internal negative control probes included in each assay were used to determine a background threshold (2 standard deviations above the mean negative control probe count value) for each sample. Background was subtracted from raw count values for each probe and counts set to 0 for all probes at or below the background threshold. Background-adjusted counts were then normalized using the functions “calcNormFactors” (“method” set to “TMM”) and “estimateDisp” (“robust” set to “TRUE”) with differentially expressed miRNAs identified using a generalized linear model-likelihood ratio test implemented in the edgeR package (version 3.18.1) for R.^25^ We filtered any miRNAs with fewer than 25% of samples expressing at 1 count per million or higher and accounted for samples from the same patient with a term in the model.

### TCGA Data Analysis

The miRNA expression values (miRNA Expression Quantification data files) for esophageal cancer samples were obtained from https://gdc.cancer.gov/. Tumor samples from patients with esophageal adenocarcinoma were selected based on histological diagnosis. Case *TCGA-L5-A4OI* was excluded because of missing annotation for why 2 miRNA quantification files were available for this case. The R package *biomaRt*^26^ in combination with the Feb 2014-Ensembl archive was used to map the gene annotations from miRBase identifiers to Ensembl gene identifiers and HGNC symbols. Only mappings with HGNC symbols related to miRNAs were retained. miRBase identifiers mapping to multiple Ensembl or HGNC symbols were removed.

### Expression-based Correlation Between miRNAs

The data were transformed using variance stabilization from the *DESeq2*^27^ R package. Preselected miRNAs were investigated for correlation among their expression values in the tumors. Expression values were averaged in case multiple Ensembl identifiers mapped to the same HGNC symbol. miRNAs with no variation across the samples were excluded. The matrix of Pearson’s correlation coefficients was clustered using hierarchical clustering with Euclidean distance and complete agglomeration as implemented in *heatmap.2*-function from the *gplots* R package.

### Methylation Analysis

Methylation data from 10 squamous and 20 Barrett’s cases were generated using Illumina (San Diego, CA) EPIC Array platform. All samples were processed through ChAMP1 program in R platform and data were normalized using BMIQ2 algorithm. The Mann-Whitney test was used for observing any differences in the methylation levels at MIR192 and MIR194-2 between squamous and Barrett’s groups. The median beta of all probes annotated to MIR192 and MIR194-2 were considered for this.

### Quantitative Real-time Polymerase Chain Reaction (qPCR)

RNA was reverse transcribed to complementary DNA using the miScript II RT Kit (Qiagen) using the manufacturer’s protocol. qPCR reactions were performed in triplicate in a 384-well plate using LightCycler 480 Instrument II (Roche, Basel, Switzerland) according to the manufacturer's protocol. Primer sequences are detailed in Supplementary Table 1.

The threshold cycle was determined by the Second Derivative Maximum method. The expression of each target was normalized relative to the geometric mean of endogenous controls. Endogenous controls for miRNA (MIR103, MIR191, MIR21) and mRNA (glyceraldehyde-3-phosphate dehydrogenase, *ACTB*, *RPS18*) targets were selected by a literature review. Consistent miRNA and mRNA endogenous control expression was validated using internal and published microarray datasets.^28^, ^29^

### Application of Selected miRNAs to Cytosponge Samples

For validation, miRNAs were selected based on having a log_2_ fold change >1 and adjusted *P* < .05 (Benjamini-Hochberg correction) in both biopsy miRNA profiling sets. In the Cytosponge set, we calculated mean fold-change differences between BE and NE Cytosponge samples as well as *P* values based on the Mann-Whitney test. For all multivariable miRNA models, we used 5-fold cross-validation using Stata version 14.0 (StataCorp LP, College Station, TX) to obtain estimates of performance criteria. Subjects were randomly assigned to 5 mutually exclusive groups with approximately equal numbers of BE cases and NE controls in each group. For a given fold, we used the 4 retained groups to model the selected miRNAs as continuous variables using logistic regression from which we estimated prediction probabilities for the group that was omitted from the fold. We repeated this prediction procedure 5 times, each time sequentially omitting a single distinct group of subjects to estimate prediction probabilities of case-control status. Prediction probabilities of ≥0.478 (87 BE/182 total) were interpreted to indicate BE case status, which was then used to estimate sensitivity, specificity, and the area under the receiver operating characteristic curve (AUC). The first multivariable miRNA model for which we assessed performance criteria included all miRNAs that replicated (were positively associated with BE) in univariate analyses in this Cytosponge sample set. Next, we assessed a reduced multivariable model selected from a stepwise logistic regression model (significance level for removal from the model = 0.1) using the total validation case-control sample set. We next assessed addition of TFF3 to this reduced model. Last, we assessed the performance criteria of a multivariable model selected using the same stepwise model to select from all miRNAs that replicated as well as TFF3. Performance criteria for all multivariable models was assessed used 5-fold cross-validation as previously described.

### In Vitro Cell Culture, Transfection, and Viability Assay

Normal esophageal squamous (NES) cells (gift from R. Souza, University of Texas Southwestern Medical Center, Dallas, TX) were cultured in a supplemented 3:1 mixture of Dulbecco’s modified Eagle’s medium/Ham's F12 medium (Invitrogen, Carlsbad, CA) as previously described.^30^ Cell numbers were determined by trypan blue cell exclusion method after 48-hour transfection.

**Supplementary Figure 1:**
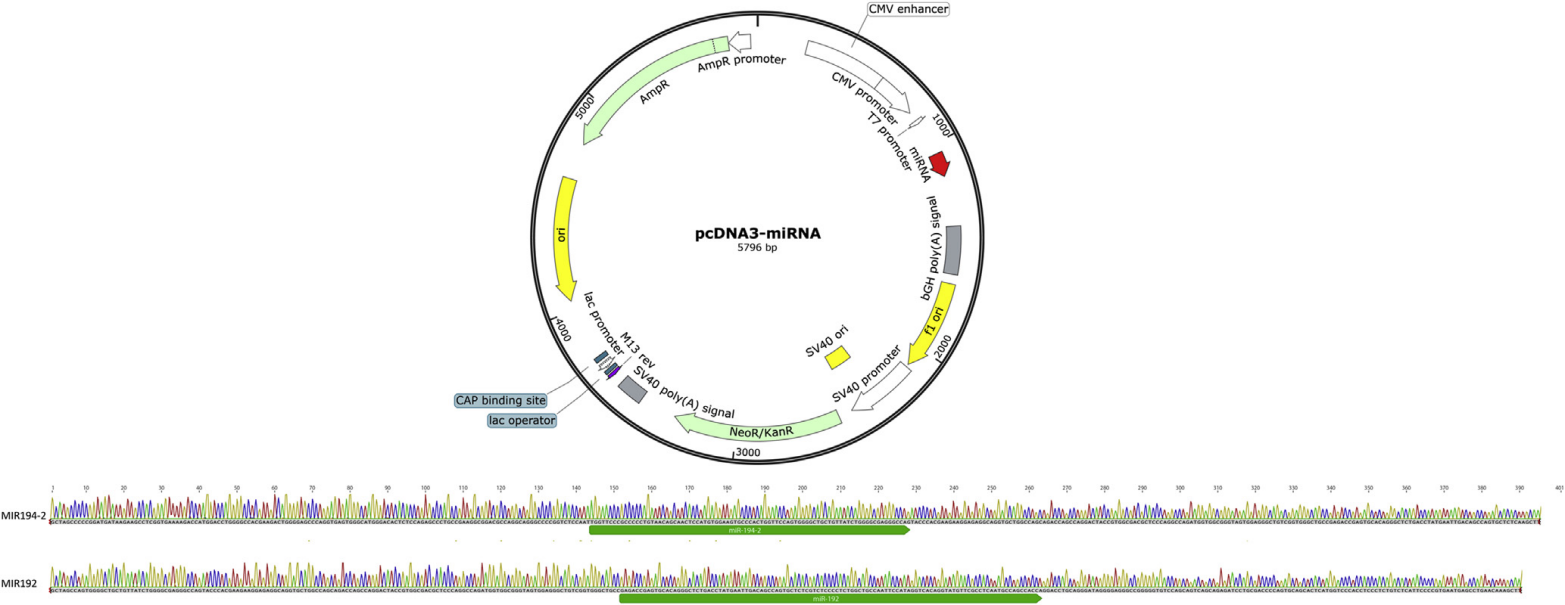
Plasmid map and sequences of MIR192/MIR194 verified by sanger sequencing.

Plasmid sequence (Supplementary Figure 1) and transfection miRNA expression plasmids were cloned by replacing the insert from a pcDNA3.1 plasmid (plasmid #21114; Addgene, Cambridge, MA) with inserts cloned by PCR (primers detailed in Supplementary Table 1). Plasmid cloning was validated by Sanger sequencing using a CMV-F primer (Supplementary Figure 1). Transfection was performed by using Lipofectamine 3000 with expression plasmid or anti-miR (miRIDIAN microRNA Hairpin Inhibitor; Dharmacon, Lafayette, CO) according to the manufacturer's protocol. All data reflect at least 2 biological replicates and refer to fold change versus empty vector, with each experiment normalized to untransfected controls, 48 hours after transfection, unless otherwise stated.

## Results

### Patient Characteristics

This study examined samples derived from 3 sets of patients with BE and NE controls as summarized in Table 1 and Figure 1. The number of samples for each tissue type in sample sets A and B were similar: samples were selected from 26 NE and 40 patients with BE (17 for BE tissues, 23 for BNE [squamous epithelium from above the Barrett’s segment]). The extracted material was compiled to form 2 NE, 2 BNE, and 2 BE pools of miRNAs for Agilent Technologies Human miRNA Microarray v1.0; 20 NE and 21 BE (BE and BNE) for Nanostring Human miRNA Expression Assay v2. To maximize the power of detection and accuracy of any findings, fresh frozen preserved endoscopically collected biopsy samples were prioritized for the profiling assays, where possible. Furthermore, matched patient tissue samples were used in Nanostring nCounter assay, which offered insights into the miRNA expression profile of BE and BNE within the same patients with BE.

**Table 1:** Summary of Patient Characteristics

Variables	Selection sample set A			Selection sample set B		Application sample set	
	NE	(B)NE	BE	NE	BE	Controls	BE cases
Numbers	26	23	17	20	21	95	87
Age, *y*, median (IQR)	56 (44–72)	66 (62–73)	63 (57–72)	60.5 (55–64)	63 (54–70)	55 (43–68)	66 (54–72)
Male: female ratio	0.73	3.6	4.67	1.86	9.5	0.98	4.8
Ethnicity: white	NA	NA	NA	20	19	89	84
Ethnicity: other	NA	NA	NA	0	2	6	3
BMI, median (IQR)	NA	26.8 (25.0–28.7)	25.6 (22.9–27.5)	28.2 (25.0–33.2)	28.0 (26.2–30.4)	27.3 (24.1–31.6)	28.4 (26.2–31.3)
Waist-to-hip ratio, median (IQR)	NA	NA	NA	0.89 (0.82–0.93)	0.95 (0.88–0.99)	0.88 (0.83–0.95)	0.95 (0.91–0.99)
Maximum M length of BE (*cm*), median (IQR)	NA	2 (1–2)	4 (4–6)	NA	6 (4–9)	NA	4 (3–7.5)
Smoking (no)	17	10	9	8	6	54	46
Smoking (ex)	1	4	1	7	13	25	28
Smoking (active)	0	2	0	5	2	14	13
Smoking (NA)	8	7	7	0	0	2	0

BMI, body mass index; IQR, interquartile range; NA, not available.

For the application of the miRNA panel, Cytosponge samples were randomly selected from cases and controls who had participated in the BEST2 study (details in methods). Cases comprised individuals with histopathologically verified BE biopsies and Cytosponge samples. For all parts of the study patients with BE were older, more likely to be male, and have higher waist-hip ratio than controls, which is consistent with known risk factor for BE^31^ (Table 1).

### Up-regulated miRNAs Detect BE in Esophageal Biopsy Samples

**Supplementary Figure 2:**
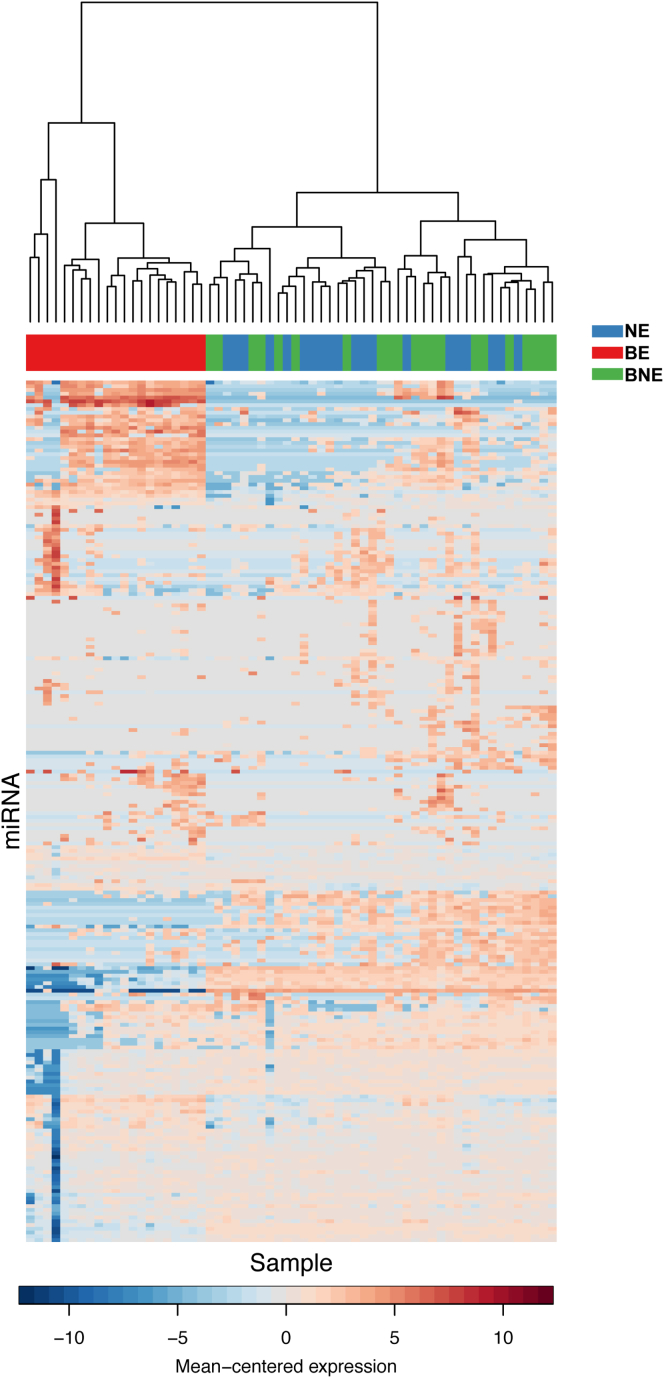
A comparison of mean-centered miRNA expression between NE (esophagus squamous tissue from normal patient), BNE (esophagus squamous tissue from BE patient) and BE (Barrett's esophagus) in Selection sample set B. Data were plotted using default values of heatmap.2 in R and then clustered with hclust (ward.D2).

Previous studies have indicated suboptimal correlations between different miRNA profiling platforms.^17^, ^18^, ^19^, ^20^, ^21^ To maximize the robustness of a miRNA signature differentially expressed in BE vs NE, we therefore performed 2 parallel high-throughput approaches (Agilent Technologies microarray and Nanostring nCounter) on 2 independent sample sets (A and B, Figure 1). Furthermore, given the high tissue specificity of miRNA profiles and the possibility of a field effect from adjacent Barrett’s, we used 2 control NE tissues. These comprised squamous tissues from healthy controls without Barrett’s (NE) as well as squamous epithelium from above the Barrett’s segment in cases (BNE) (see study design Figure 1 and Table 1). The miRNA profile of BNE and NE samples clustered together (Supplementary Figure 2) and there were 4 miRNAs (MIR451, MIR144, MIR191, MIR375) that showed differential expression between NE and BNE samples (Supplementary Table 2). However, the fold changes were modest. In view of the similarities between BNE and NE miRNA expression profiles, these groups were combined for subsequent analyses.

**Figure 2:**
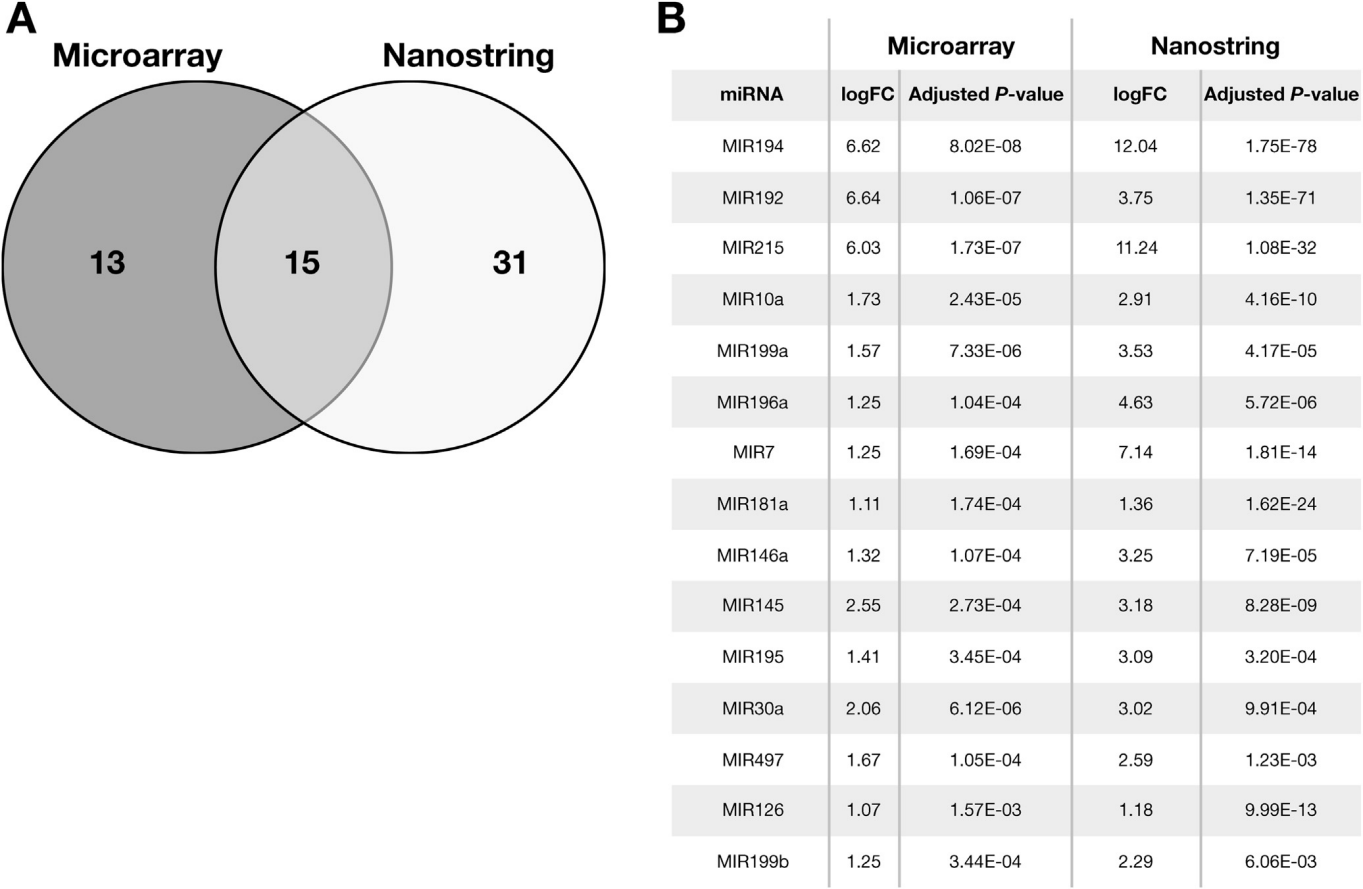
Up-regulated miRNAs from cross-platform analysis. (*A*) Venn diagram showing up-regulated miRNAs detected by Agilent microarray and Nanostring profiling in Selection sample sets A and B respectively. Detailed fold changes for each up-regulated miRNA listed in Supplementary Tables 2 and 3. Fifteen consensus miRNAs were determined by cross-referencing of miRNAs detected by each platform. Log of fold changes (logFC) and adjusted *P* values of these 15 miRNAs are listed in (*B*), ranked by mean of adjusted *P* value.

When comparing squamous (BNE + NE) and Barrett’s (BE) samples, miRNA Microarray (Agilent Technologies) expression analysis in sample set A identified 28 up-regulated miRNAs of 470 total measured (Supplementary Table 3), whereas Nanostring nCounter Human v2 microRNA Expression Assay analysis in sample set B identified 46 up-regulated miRNAs of 800 (Supplementary Table 4). Cross-referencing of up-regulated miRNAs (defined as log fold change >1 and adjusted *P* < .05) identified 15 miRNAs significantly up-regulated in both sample sets (Figure 2).

### Novel miRNA Panel as Effective Biomarkers for Noninvasive BE Diagnosis

**Figure 3:**
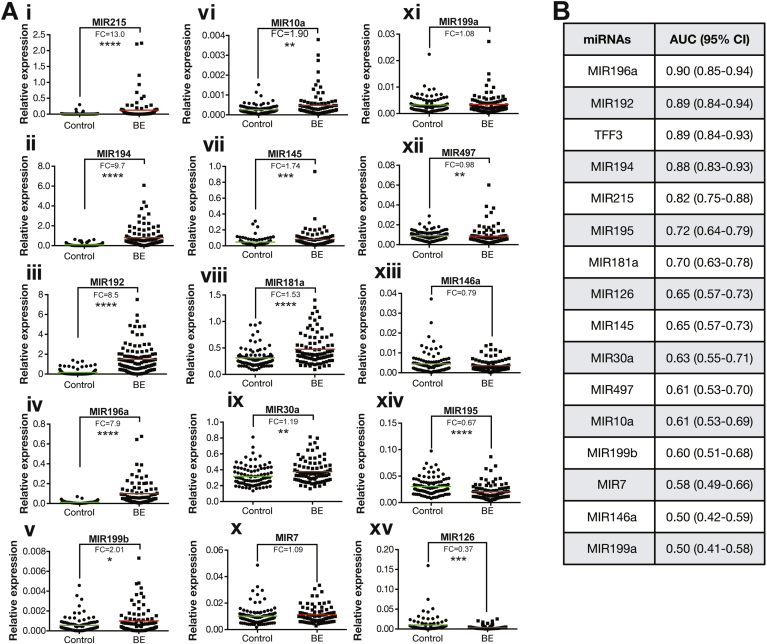
Validation of up-regulated miRNAs in case-control Cytosponge sample set. (*A i*–*xv*) Relative miRNA expression determined by qPCR Fold changes (FC) and mean (colored line) are presented for each miRNA. Significance determined by Mann-Whitney test: *P < .05; **P < .01; ***P < .001; ****P < .0001. (*B*) AUC and 95% CI for each miRNA and TFF3 were calculated using validation qPCR results.

To assess the diagnostic performance of these 15 miRNAs using Cytosponge samples, their expression was examined by qPCR in a distinct set of 95 control and 87 BE samples randomly selected from the BEST2 Cytosponge study.^6^ On univariate analysis, MIR215, 194, 192, and 196a were significantly (*P* < .0005) and highly (fold changes of relative expression of 13.0, 9.7, 8.5, and 7.9, respectively) up-regulated in case vs control Cytosponge samples (Figure 3*A i*–*iv*) with AUC of 0.82 (95% confidence interval [CI] 0.75–0.88), 0.88 (95% CI 0.83–0.93), 0.89 (95% CI 0.84–0.94), and 0.90 (95% CI 0.85–0.94), respectively (Figure 3*B*). This increased expression pattern for BE vs NE was also replicated for a further 7 of the 15 miRNAs on univariate analysis: 199b, 10a, 145, 181a, 30a, 7, and 199a (fold changes >1 between BE and control patients, Figure 3*A v*–*xi*); however, miRNAs 195, 126, 497, and 146a failed to replicate in this Cytosponge set of cases and controls (fold change <1 between BE and control patients, Figure 3*A xii*–*xv*). These 4 miRNAs were, therefore, not considered for multivariable models.

The AUC of a model including age, sex, race/ethnicity, body mass index, waist-to-hip ratio, and smoking status was 0.71 (95% CI 0.64–0.77), which dramatically improves when the predictive tissue biomarkers are added (Table 2). Five-fold cross-validation of a multivariable biomarker model that included the 11 validated miRNAs provided an AUC of 0.87 (95% CI 0.82–0.92) with a sensitivity 83.9% and specificity 90.5% (Table 2) using a predicted probability threshold of ≥0.478 (87 BE/182 total) to assign BE case status (see Methods). Stepwise selection in the total set followed by 5-fold cross-validation suggested that a subset of miRNAs (MIR7, 30a, 181a, 192, 196a, 199a) slightly improved the AUC to 0.89 (95% CI 0.84–0.93) with 86.2% sensitivity and 91.6% specificity (Table 2). Inclusion of TFF3 improved the AUC to 0.92 (95% CI 0.88–0.96), although statistically there is no significant difference with or without TFF3 (Table 2). Stepwise selection of the 11 validated miRNAs and TFF3 retained just 3 miRNAs (MIR192, 196a, and 199a) as well as TFF3 and 5-fold cross-validation of this model provided an AUC of 0.93 (95% CI 0.90–0.97) with sensitivity 93.1% and specificity 93.7% (Table 2).

**Table 2:** AUCs and 95% CIs of Stepwise Selected Biomarker Models Using 5-fold Cross-validation

Model details	Predictors	AUC (95% CI)	AUC (95% CI) with risk factors for BE
Risk factors for BE	Age, sex, ethnicity, smoking, body mass index, waist-hip ratio	0.71 (0.64–0.77)	—
All miRNAs that univariately positively predicted BE in Cytosponge application	MIR7, 10a, 30a, 145, 181a, 192, 194, 196a, 199a, 199b, 215	0.87 (0.82–0.92)	0.84 (0.79–0.89)
Stepwise selection of miRNAs from initial model	MIR7, 30a, 181a, 192, 196a, 199a	0.89 (0.84–0.93)	0.88 (0.83–0.93)
Above model plus TFF3	MIR7, 30a, 181a, 192, 196a, 199a plus TFF3	0.92 (0.88–0.96)	0.92 (0.88–0.96)^a^
Stepwise selection of miRNAs from initial model and TFF3 (3 miRNAs and TFF3)	MIR192, 196a, 199a, plus TFF3	0.93 (0.90–0.97)	0.91 (0.87–0.95)

a Model did not include smoking due to failure to converge.

### Epigenetic Alteration in BE Could Contribute to Aberrant Coexpression of Cluster miRNAs

**Figure 4:**
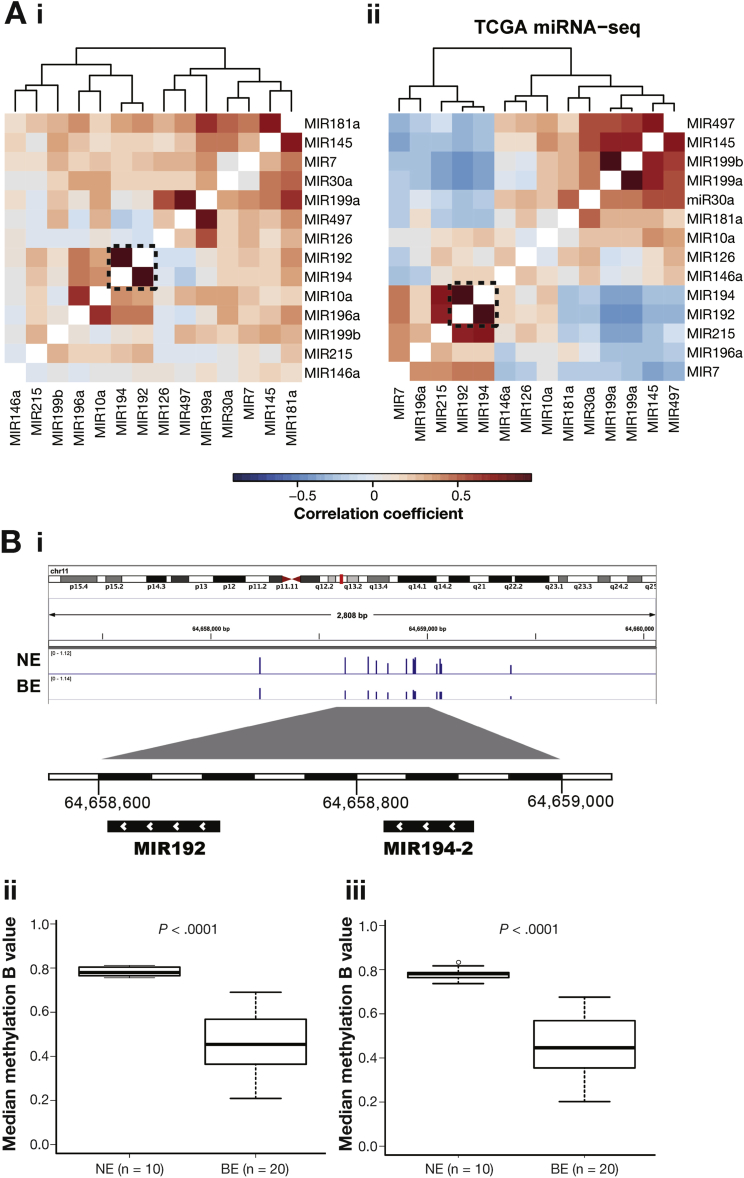
Coexpression of MIR192/194 and hypomethylation found in the promotor region of miRNA cluster MIR192-194-2. (*A*) Heatmap showing the Pearson correlation coefficient (color key) between 15 consensus up-regulated miRNA expressions based on validation data from this study (*A i*) and TCGA esophageal adenocarcinoma miRNA sequencing data (*A ii*). Dendrograms show the hierarchical clustering based on the complete linkage method and Euclidean pairwise distance. Genomic region of cluster MIR192-194-2 shows DNA methylation probe peaks in the promoter region (<1 kb) in NE and BE samples (*B i*). Median methylation beta values were plotted for MIR192 and MIR194–2 (*B ii*). Significance was determined by Mann-Whitney test.

Interestingly, 3 of the most significantly up-regulated miRNAs are from 2 miRNA clusters, MIR192-194-2 (11q13.1) and MIR215-194-1 (1q41, intron of *RNU5F-1* and *IARS2*), which have been reported to respond to p53 activation.^32^ Although MIR194-1 and MIR194-2 are located on different chromosomes, they share an identical mature sequence and target the same type of mRNAs. Clustered miRNAs usually share a similar expression pattern^33^ and the correlation matrix based on the case-control sample set revealed the coexpression of MIR192 and MIR194 (*r* = 0.787, Figure 4*Ai*). This observation can be replicated using TCGA esophageal adenocarcinoma miRNA sequencing data (n = 88) as independent data set (*r* = 0.945, Figure 4*Aii*).

To understand the increased coexpression of MIR192 and MIR194-2 in BE, we searched for genomic and epigenetic alterations. By using whole genomic sequencing data from our previous genomic studies,^8^, ^34^, ^35^ no recurrent somatic mutations were found in the known regulatory regions of MIR192-194-2. Interestingly, the promoter regions of MIR192-194-2 (<1 kb from transcription starting site) were highly methylated in the NE samples (n = 10), whereas a significant hypomethylation (*P* < .0001) was found in the distribution of methylation intensity in the BE samples (n = 20), which is well-known to be correlated with target gene overexpression^36^ (Figure 4*B i*–*iii*).

### MIR194 Dependent Signaling Prompts Esophageal Cell Growth In Vitro

**Supplementary Figure 3:**
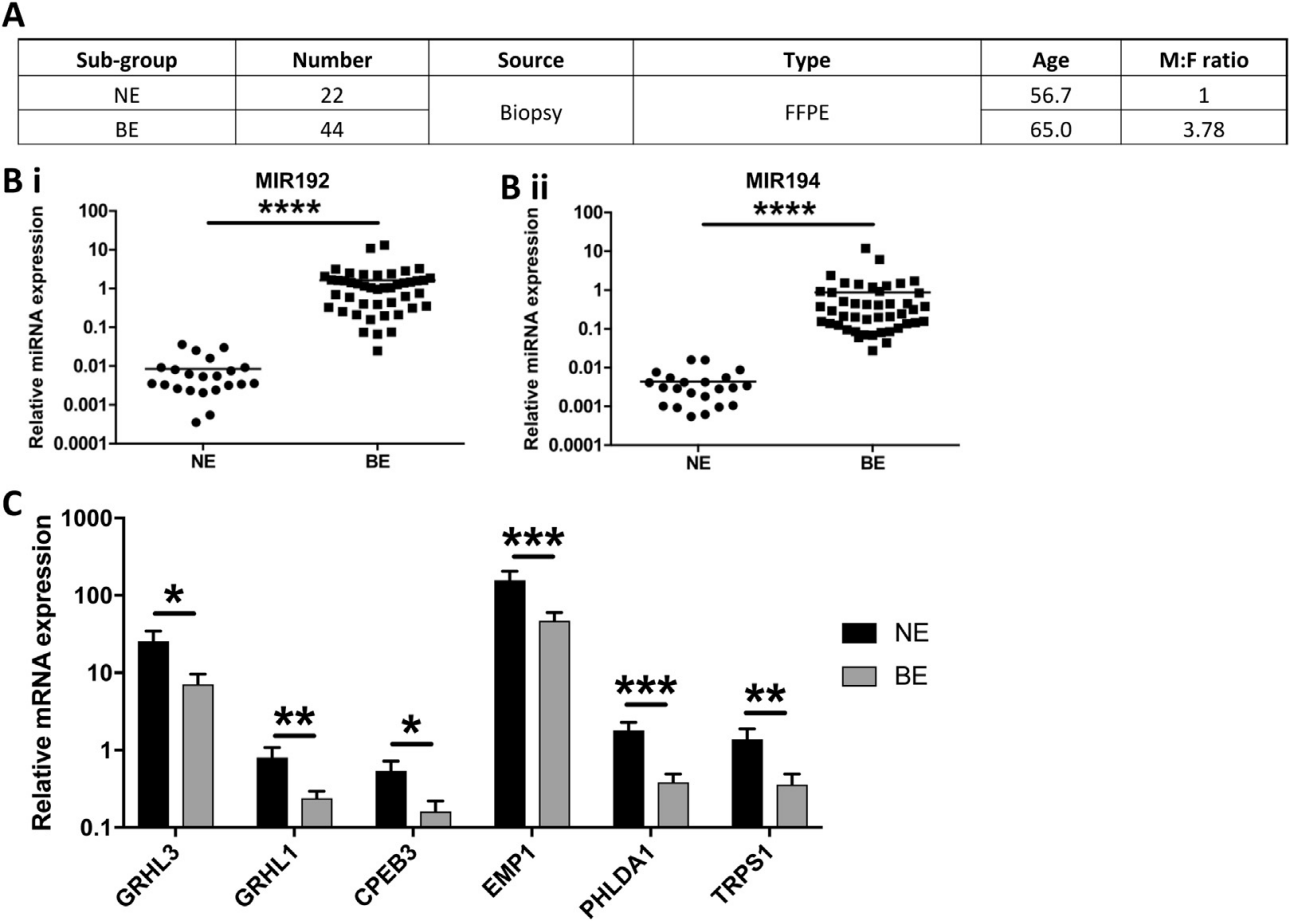
Upregulation of putative mRNA targets of MIR192 and MIR194 in NE versus BE biopsy samples (*A*) Summary of biopsy sample set. (*B, C*) Relative expression of MIR192 (B i) and MIR194 (*B ii*) and their putative targeted mRNAs (*C*) were determined by qPCR Error bars show standard deviation of the mean: ∗*P* < .05; ∗∗*P* < .01; ∗∗∗*P* < .001; ∗∗∗∗*P* < .0001.

Next, we set out to examine the role of up-regulated MIR192/194 through their target mRNAs in BE. We predicted targets of miRNAs 192 and 194 using the TargetScan 7.1 algorithm, which searches for conserved 3′ untranslated region (UTR) sites that match the seed region (nucleotides 2–7) of each miRNA.^37^ We also incorporated mRNAs down-regulated >20% following MIR192 transfection using data from a published microarray dataset to populate our list of predicted targets.^38^ We hypothesized that true targets of these miRNAs would be down-regulated in BE vs NE biopsy samples. Using recent microarray datasets,^28^, ^39^ 53 such putative targets were identified and, following a literature review to prioritize targets with known tumor suppressor roles in cancer, 6 were selected for further validation (Supplementary Table 5). qPCR confirmed down-regulation of all of these targets, and the increased expression of MIR192 and MIR194 (Supplementary Figure 3).

**Figure 5:**
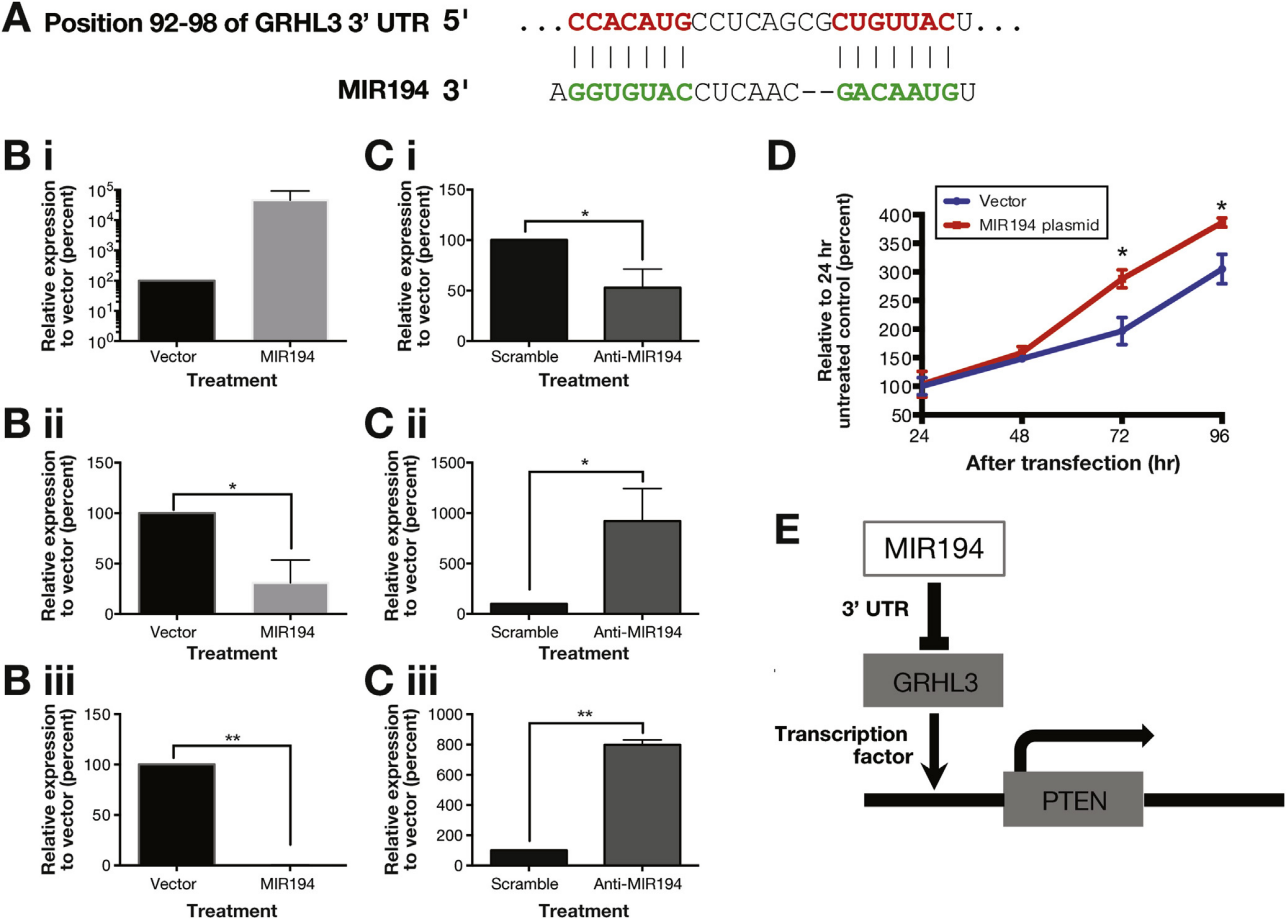
Dysregulated MIR194 expression drove cell proliferation in vitro through MIR194-GRHL3-PTEN axis. (*A*) Genomic alignment of GRHL3 mRNA 3′ UTR with MIR194. Normal esophageal cell line NES were transfected with either MIR194 plasmid (*B*) or anti-MIR194 (*C*). Relative expression of MIR194 (*i*), GRHL3 (*ii*), and PTEN (*iii*) in NES cell line was examined by qPCR Error bars show standard deviation of the mean: *P < .05; **P < .01. (*D*) Cell growth was determined by trypan blue exclusion assays every 24 hours for a period of 96 hours after MIR194 transfection relative to vector control. Error bars show standard deviation of the mean: *P < .05. (*E*) Diagram shows MIR194-GRHL3-PTEN cascade network.

**Supplementary Figure 4:**
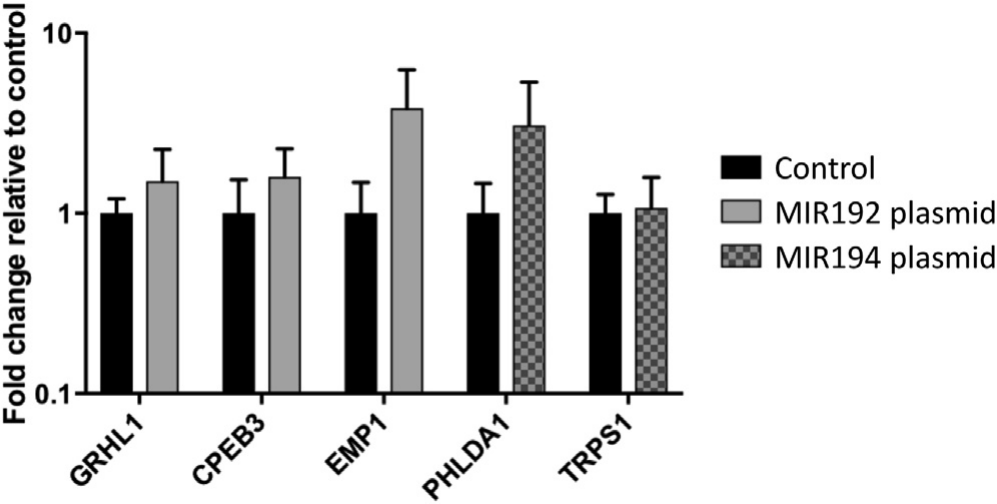
Expression of putative mRNA targets in normal esophageal cells with transit over expression of MIR192 and MIR194 Normal esophageal cell line NES was transfected with MIR192 and MIR194. Relative expression of putative targeted mRNAs of MIR192 and MIR194 were determined by qPCR after 48 hours of transfection.

To demonstrate repression of putative miRNA targets in vitro, cell line NES derived from NE^30^ was transfected with MIR192 or MIR194 expression plasmids. *GRHL3*, one putative target mRNA of MIR194 (Figure 5*A*) was significantly down-regulated on MIR194 overexpression (fold change >3, Figure 5*B i*–*ii*), whereas the other 5 miRNA targets examined were not significantly repressed on transfection (Supplementary Figure 4). To further characterize this relationship, NES cells were transfected with antisense oligonucleotides against MIR194 (anti-MIR194). This was associated with concurrent down-regulation of MIR194 and up-regulation of *GRHL3* compared with control anti-miRNA (Figure 5*Ci*–*ii*). Comparative analysis of the *GRHL3* 3′ UTR across vertebrates using TargetScan 7.1 showed that it contains 2 conserved 7mer-m8 binding sites for MIR194 (Figure 5*A*).^40^ In summary, MIR194 negatively regulated *GRHL3* expression both in silico and in vitro.

GRHL3 is known to activate *PTEN* transcription by binding to a conserved site in the *PTEN* promoter.^41^ Transfection of MIR194 in NES cell line was associated with significant repression of *PTEN* expression (Figure 5*Biii*). In contrast, suppression of MIR194 by anti-MIR194 leads to up-regulation of *PTEN* (>7-fold) (Figure 5*Ciii*). Furthermore, consistent with the function of PTEN as a negative regulator of growth signaling,^42^ *PTEN* down-regulation on MIR194 overexpression was associated with significantly enhanced cell growth in vitro at 72 hours following transfection (Figure 5*D*). Taken together, our findings highlight the regulation of MIR194 on esophageal cell growth through the MIR194-GRHL3-PTEN axis (Figure 5*E*).

## Discussion

Using patient biopsy and Cytosponge samples, this study identified a panel of miRNAs that are differentially expressed in BE vs NE and accurately diagnosed BE using Cytosponge samples. We demonstrated that these miRNAs may have a functional role in BE etiology, whereby increased expression of MIR194 drives proliferation in an in vitro NE model through the MIR194-GRHL3-PTEN regulatory network.

To improve the profiling signals and reproducibility of miRNA discovery platforms,^17^, ^18^, ^19^, ^20^, ^21^ we used 2 profiling methods in pathologically verified biopsy samples. A panel of 15 up-regulated miRNAs in BE biopsy samples were identified, as well as some lesser known and novel candidates (MIR196a, 199a/b, 7, 181a). It is reassuring that this study identified some miRNAs, including MIR192, MIR194, and MIR215, that have been shown to be up-regulated previously.^43^, ^44^, ^45^ Stepwise selection was used to identify the minimum panel with the maximum AUC. A subset of miRNAs (MIR7, MIR30a, MIR181a, MIR192, MIR196a, MIR199a) provided an AUC of 0.89 with 86.2% sensitivity and 91.6% specificity. A logistic regression model with stepwise selection that included TFF3 provided an optimal panel comprising MIR192, MIR196a, and MIR199a and TFF3 with an AUC of 0.93, the greatest sensitivity of 93.1%, and greatest specificity of 93.7% (Table 2). It was interesting to note that MIR199a was retained in the final panel despite an individual AUC of 0.50 (Figure 3). There was no effect modification between MIR199a and MIR192, MIR196a, or TFF3. According to our a priori rules, MIR199a was retained in the stepwise multivariable model based on a low *P* value. However, excluding it from the 5-fold cross-validation has no material effect on the AUC (0.93, 95% CI 0.89–0.97). It should be noted that the previously reported TFF3 accuracy data were ascertained from a prospective trial with larger sample numbers than the current study.^6^

From the perspective of clinical translation, an miRNA assay could be readily adapted to a high-throughput setting amenable for large-volume screening, whereas TFF3 relies on the preparation of a cell block and histopathological and immunohistochemical assessment by an expert. Here we demonstrate that an miRNA panel (MIR7, MIR30a, MIR181a, MIR192, MIR196a, MIR199a) can provide a very similar accuracy with an AUC of 0.89 (86.2% sensitivity and 91.6% specificity) compared with TFF3 alone with an AUC of 0.89 (83.9% sensitivity and 93.7% specificity) when applied to the same sample set (Figure 3). Furthermore, the AUC achieved for a combination of TFF3 and miRNAs is not statistically improved and the laboratory processing for a combination would be more complex. miRNA expression analysis could be performed using an automated pipeline with objective quantitation. Diagnostic miRNAs also could be quantitated in parallel with other nucleic acid biomarkers for risk stratification.^7^, ^8^

However, this study does have limitations. Although development of the Cytosponge predictive algorithm was based on selection of the most promising miRNA candidates from independent sample sets by 2 profiling platforms, some miRNAs were exclusively assessed on one of the profiling platforms, thus precluding their selection for the subsequent Cytosponge samples. In addition, the model performance may be somewhat overoptimistic given the lack of an external independent Cytosponge sample set. Given the complexity of the miRNA-mRNA network and its epigenetic regulation, further study is required to elucidate the precise role of these miRNAs in the pathogenesis of BE.

The correlation matrix of the top 15 miRNAs in the BE and NE dataset suggested that MIR192 and 194 may be coregulated. This high correlation (*r* = 0.78) was also evident in TCGA esophageal adenocarcinoma data (*r* = 0.945). MIR192 and MIR194 are located within a single 300-bp miRNA cluster at 11q13.1. Unlike cluster MIR215-194-1, the MIR192-194-2 cluster is not within an intron of a host gene, suggesting a less complex transcription regulation.^11^ Some initial efforts were made to understand the cause of these up-regulated miRNAs by examining genomic and epigenetic alterations, and we identified hypomethylation distribution in the promotor region of the cluster MIR192-194 that could explain the up-regulation of clustered miRNAs.

We hypothesized that some or all of these miRNAs may be relevant in the pathogenesis of BE by repression of other genes. Using a combination of in silico and in vitro approaches to find and validate putative targets, we found that MIR194 could repress *GRHL3* likely through its conserved binding site in the 3′ UTR of *GRHL3*. *GRHL3* positively regulates the tumor suppressor *PTEN,* whereas *GRHL3* knockout results in squamous cell carcinoma development in vivo associated with activation of phosphatidylinositol-3-kinase signaling.^41^ PTEN functions to negatively regulate signaling in the phosphatidylinositol-3-kinase pathway by dephosphorylating PIP_3_ to prevent activation of AKT and mammalian target of rapamycin, thus inhibiting cell survival and proliferation.^46^ In line with evidence that BE is associated with increased proliferation,^47^ we demonstrated that MIR194-mediated GRHL3 repression associated with reduced PTEN expression and increased proliferation in vitro. Previous studies revealed that the loss of PTEN expression is an independent negative prognostic factor in esophageal adenocarcinoma.^48^

In conclusion, we have identified a panel of up-regulated miRNAs that can diagnose BE in biopsy and nonendoscopic Cytosponge samples. Hypomethylation found in the promoter regions of these biomarker miRNAs could contribute to the dysregulation of miRNAs with phenotypic consequences through their target mRNA network. Further work is required to apply this miRNA strategy to a prospective Cytosponge trial in the primary care setting, and explore the feasibility of a high-throughput, automated platform that could potentially be combined with other nucleic acid biomarkers for risk stratification.
