# Supplementary material for: Selection and Application of Tissue microRNAs for Nonendoscopic Diagnosis of Barrett’s Esophagus
Source: Gastroenterology. 2018 Sep;155(3):771–783.e3. doi: 10.1053/j.gastro.2018.05.050 (PMC6120784; doi:10.1053/j.gastro.2018.05.050)
Supplement: Legends for Supplementary Tables 1–5 [file mmc6.pdf]

SupTable 1: Differentially expressed miRNAs between NE and BNE from Selection sample set A and B

SupTable 2: Differentially expressed miRNAs from microarray profiling of Selection sample set A

SupTable 3: Differentially expressed miRNAs from Nanostring nCounter profiling of Selection sample set B

SupTable 4: Putative miRNA targets of MIR192 and MIR194 identified by cross-referencing predicted targets with miRNAs known to be downregulated in BE.

SupTable 5: Primer sequences used for qRT-PCR and PCR
